# Relational anonymity in reducing the harms of illicit drug use: accounts of users of dark web- and street-based services in Finland

**DOI:** 10.1186/s12954-024-01139-y

**Published:** 2024-12-21

**Authors:** Johanna Ranta, Teemu Kaskela, Juha Nurmi, Teemu Ruokolainen, Gillian W. Shorter

**Affiliations:** 1 Faculty of Social Sciences, Tampere University, Tampere, 33014 Finland; 2 A-Clinic Foundation, Helsinki, 00510 Finland; 3 Faculty of Information Technology and Communication Sciences, Tampere University, Tampere, 33720 Finland; 4 Aalto University, Espoo, 02150 Finland; 5 Drug and Alcohol Research Network, Queen’s University Belfast, Belfast, BT9 1NN UK

**Keywords:** Anonymity, Drug use, Harm reduction, Outreach work, Street-based services, Dark web-based services, Tor network, Account, Qualitative analysis, Stigma

## Abstract

**Background:**

Protecting individual anonymity is a common practice in harm reduction (HR), as it can mitigate the fears that may prevent people from accessing services. Protecting anonymity usually means applying for services with a pseudonym. However, anonymity protection practices have diversified in current HR environments, for example, on the streets or in the Tor network, which relies on technology to guarantee exceptionally strong anonymity. Despite its importance, the individual’s need for anonymity when seeking help to reduce drug-related harm has been underexplored.

**Methods:**

The research contexts included four street- and dark web-based HR services in Finland. The data consisted of service user interviews and naturally occurring conversations in the Tor network. We focused on service users’ accounts of their need for anonymity and applied the concept of relational anonymity, acknowledging that wider contextual relations intertwine with situational needs for anonymity. We asked: What kinds of needs for anonymity do service users express when discussing seeking help to reduce drug-related harm? How do service users account for their need for anonymity when seeking such help? To which kinds of contextual relations are these accounts attached?

**Results:**

We identified connections between the accounts of the need for anonymity and various contextual relations: (1) excusing the need for anonymity by referring to societal relations: blaming Finnish society for stigmatising attitudes and exclusionary practices; (2) excusing the need for anonymity by referring to service system relations: blaming the service system for the risk of negative consequences from recording the use of illicit drugs; (3) justifying and excusing the need for anonymity by referring to personal relations: appealing to personal situation, feelings and experiences.

**Conclusions:**

The need for anonymity reflects problematic societal relations, in which the stigma towards drug use is strong. The service users’ accounts were motivated by rational actions to avoid possible sanctions and the perceived abuse of power in Finnish society and services, which the service users deemed to have various negative consequences in their lives. Societies should promote cultural atmospheres and information sharing practices where anonymity is not needed, but services that protect anonymity are crucial in the current societal conditions.

## Background

In Finland, protecting individual anonymity has been an essential practice in harm reduction (HR) services throughout their history [1]. The practice has traditionally involved the possibility of accessing services (e.g. needle exchange units) using a pseudonym without having to provide a name or a social security number. Anonymity may facilitate service access and assuage fears about applying for needed services [see 2]; people who use drugs may face stigma, mistreatment and other barriers when seeking help from social and healthcare services, which may decrease trust and service uptake [3–5].

Goffman [6] defines stigma as desecration expressed by other parties (such as other citizens in society or service professionals) that degrades and tarnishes a person’s value and leads to their unwanted separation from others. Ultimately, this can lead to self-stigmatisation, in which the feeling of ​​not deserving a respected social status becomes a personal belief [7]. Stigmatising people who use drugs is not unusual in Finland [4], where the national drug legislation is based on total prohibition, with the production, trade, possession and use of drugs defined as criminal activities that may result in prison sentences [8]. Categorising drug users as ‘criminals’ may result in their not being recognised as full members of society, thus limiting their access to assistance. Some people face multiple stigmas and social tensions, for example, if they are parents who use illicit drugs, which can make seeking help even more difficult [4].

The importance of anonymity in seeking help for drug-related harm is not new. In Narcotics Anonymous (NA) groups, anonymity promotes equality among group members by hiding individuals’ social status, backgrounds and life situations [9]. Although HR’s goals and practices differ from those of NA, protecting anonymity aligns well with HR principles related to human rights. It reinforces people’s freedom of choice and right to self-determination and reflecting a pragmatic, non-moralising approach; people are supported in their presenting needs regardless of their name or history with drugs [10–12].

Over the past decade, public and private health and social care organisations in Finland have promoted information sharing by recording individuals’ health and social data in a single information system. Third sector HR services have, in turn, diversified their anonymity protection practices. The implementation of anonymity has changed in response to HR services’ expansion from traditional buildings to, for example, street- and web-based outreach work [13–15]. The level of anonymity may be stronger online than in person, as people can conceal their face and voice. However, public places can also offer needed identity protection when individuals do not wish to enter the door of a physical needle exchange unit.

Recently, the HR community has expanded the implementation of anonymity by using identity-protecting instant messaging applications, such as WickrMe and Session. This approach relies on trust in technology to guarantee anonymity. In another innovation, Finnish HR professionals provide support to people who use drugs in the online forums of the Tor (The Onion Router) network [12]. Internationally, similar HR encounters have occurred mostly among peers [16–18]. Tor facilitates anonymous communication over the internet by providing an easy-to-use web browser that directs web traffic through a volunteer-operated worldwide network consisting of thousands of relays that obscure a user’s location and IP address. This shields against network surveillance or traffic analysis, making it the most popular tool for those seeking to maintain anonymity online (5 million daily users in 2024 [19]). Tor’s anonymity provides protection for some individuals who use illicit drugs and might not otherwise seek assistance because they fear the consequences of being identified [12].

The present article explores various needs for anonymity in current HR environments. Although securing anonymity has been identified as an important practice in HR services [20, 21], the situational and individual meanings of anonymity have been underexplored. This perspective is relevant, particularly in European countries whose drug policies prioritise criminalisation whilst simultaneously providing HR services [see 10, 11]. We analyse the meanings of anonymity for the users of street- and dark web-based HR services in Finland by asking (1) what kinds of needs for anonymity do service users express when they discuss seeking help to reduce drug-related harm, (2) how service users account for their need for anonymity when seeking such help and (3) to which kinds of contextual relations these accounts are attached.

To understand how service users explain their need for anonymity in their current living circumstances, we focus on their *accounts* [22]. Furthermore, we apply the concept of *relational anonymity*, acknowledging that contextual relations, such as those of a given society or service system, intertwine with situational needs for anonymity [see 23]. As in other frameworks applied to the harms associated with drugs (e.g. the Risk Environment framework [24]), we acknowledge the role of factors beyond the individual. Thus, we hypothesise that an individual’s need for anonymity is a consequence of broader social circumstances and may not necessarily be present in different social contexts.

## From technological anonymity to situational needs to protect privacy: a relational approach

The concepts of privacy and anonymity have had several meanings and definitions over time. Privacy has evolved since its conception as the ‘right to be let alone’ [25]. Originally, the concept of *anonymous* was defined as ‘one whose name is unknown’ [26]. More recent dictionary definitions of *anonymity* have emphasised namelessness, impersonality and lack of outstanding or unusual characteristics [26]. Behavioural science, in turn, argues that anonymity can be viewed from three perspectives: (1) identity protection (withholding unique identifiers, including names), (2) visual anonymity (being unseen in communication) and (3) action anonymity (in which the content and existence of actions are unknown to others), suggests that different types of anonymity have different implications [27]. Anonymity also has other summarising definitions and conceptualisations, including interpersonal disconnectedness, wherein an individual cannot be identified [27, 28]. The legal understanding remains clear: someone is anonymous if not currently identified [28].

### Online anonymity, Tor and pseudonymity

Online, anonymity is more than a component of user privacy; it is a multidimensional concept crucial to the security of many online activities, without which people would be vulnerable to eavesdropping [29]. Nurmi [30] differentiates between anonymity and pseudonymity in the Tor network, explaining that anonymity allows users to control who knows their identity, preventing adversaries from discovering it, whereas pseudonymity involves using a persistent anonymous identity, such as a nickname, which enables recognition among users. Silva and Reed [28] argue that the internet has transformed anonymous communication; online environments easily compromise anonymity through unique identifiers. They note that discussions of anonymity often overlook its complexity, as users who share information online may be identifiable later when technology evolves and multiple sources may be linked to identifying someone. This differs from the offline realm, where these connections are less likely or are prone to recall bias. Thus, the desire for anonymity cannot guarantee an absolute right to remain anonymous forever, nor should we consider there to be a reasonable expectation of it. It is not a binary on or off [28]. 

### Relational anonymity

We developed the concept of *relational anonymity* to understand the complex and situational nature of the need for anonymity. First, what is deemed a sufficiently anonymous identity may vary depending on individual people and situations. For some people using drugs, the only acceptable option is to aim for technological untraceability; for others, anonymity means being faceless, voiceless or using a pseudonym. Second, we base our perspective on social psychological research traditions that view people’s agency—their ability, capacity and power to act and make choices—as intertwined with human interactions and broader social relations [31]. We approach the need for anonymity as an element related to agency; as being *contextually constructed and changing over time and place*.

We employ a framework used previously to investigate the agency of service users and workers in Finnish HR services as linked to a context-dependent network of relations [23]. We address the need for anonymity by examining the relations intertwined with the agency of participants in HR encounters in the above-mentioned study [23]: (1) *societal relations*, e.g. global, national or local politics and values related to drug use; (2) *service system relations*, e.g. factors related to the local service system; (3) *personal relations*, e.g. relationships with HR workers and other individuals; and (4) *spatial relations*, e.g. meeting places in HR services. In addition, these intertwine with (5) *time relations*, e.g. time-bound political strategies or service users’ previous experiences of services and their consequences for current or future service choices.

## Methods

### Research contexts

The data were collected in four HR services in Finland, which aim to reach and encounter people who use drugs and do not use other health and social services for their drug-related issues. Two are dark web-based services (DWBSs). The workers meet service users on online platforms that offer technological anonymity, such as public dark web discussion forums that address the harm of illicit drug use or through text-based instant messaging applications, such as WickrMe and Session. One DWBS focuses mainly on health counselling (e.g. giving advice on how to avoid infections and inject/use drugs as safely as possible). The other DWBS offers social counselling (e.g. providing individual support in accessing services or other matters related to social situations), with the choice of online or face-to-face meetings.

The other contexts are street-based services (SBSs) providing outreach work in a Finnish city. The first SBS concentrates on homelessness, aiming to assist drug-using homeless individuals with housing-related issues, such as finding a flat or receiving the necessary services and social security. The second SBS provides social and health counselling, addressing a diverse range of social and health issues associated with drug use, e.g. supporting people to receive needed health and social services, conducting screening for blood-borne viruses and providing clean injection equipment to individuals who cannot access physical needle exchange units. SBS users are mostly encountered face to face in public streets, parks, parking lots, shopping centres or their homes, but they can also contact workers using social media applications, such as WhatsApp and Instagram, or instant messaging applications (e.g. WickrMe, Session) that secure anonymity.

These services share the priority of honouring service users’ wishes regarding where they would like to access the service. The forms of anonymity directly influence the choice of meeting places. Additionally, these services do not record data on service users in the Finnish shared information system (OmaKanta) as do, for example, public healthcare organisations. This prevents official institutions from using information about service use: people who use drugs have been found unwilling to be registered [32] and reluctance to use services that share information with surveillance institutions [33, 34]. The SBSs have their own information systems, which store brief records of encounters with people under pseudonyms, a practice common in HR services nationwide. The primary objective is to gather data on the number of encounters and type of work performed. The DWBSs provide records only to their funders based on the number of contacts and the reasons for contacting the service. The data are not recorded with pseudonyms, so service users’ encounters cannot be combined.

### Data

The data comprise 29 encounters with 28 service users in Finland in 2018–2023. The first author conducted thematic interviews (13 interviews, 617 min in total) that included questions on experiences with HR services, especially meeting places and interactions with HR workers. One question concerned service users’ opinions on and experiences with anonymity when seeking help to reduce drug-related harm, but some interviewees mentioned anonymity frequently. Our data also include naturally occurring DWBS encounters (16 messages) in the Tor network online forum analysed in our earlier study [12]. We found that people quite often spoke there about the need for anonymity. We analyse those messages from service users to HR workers related to the need for anonymity when applying for services.^1^ We describe the data corpus in Table 1.

**Table 1:** The contexts and data of interviews and naturally occurring HR conversations with 28 service users

Context	Type of data	Amount of data	Place of data production	Period
Street-based services A. Social and health counselling B. Homelessness work	Service user interviews (services A & B)	10 (1 pair & 9 individual = 11 service users)	Service facilities, cafeterias, outdoor locations, a service user’s home, a hospital	2022–2023
Dark web-based services C. Health counselling D. Social counselling	Service user interviews (service C)	3 (all individual)	Regular phone call, WickrMe (chat), Session (voice call)	2022–2023
	Online conversations (services C & D)	16 messages (from 14 service users)	Finnish discussion forum Torilauta in the Tor network	2018–2020

### Data analysis

The analysis aims to understand the various meanings of anonymity for people who use drugs when accessing social and healthcare services to reduce drug-related harm. Through the concept of relational anonymity, we approach the individual need for a given kind of anonymity as being connected to wider contextual relations [23]. Table 2 outlines the process of analysis.

**Table 2:** The process of analysis to understand service users’ accounts of the need for anonymity

Phase		Aim	Findings
1.	ATLAS.ti coding	To recognise all the episodes in which service users’ needs for anonymity were discussed	A total of 53 episodes (37 in interviews, 16 in naturally occurring online conversations)
2.	ATLAS.ti coding	To categorise the themes to which the need for anonymity was connected	Compiled in the results chapter (Table 3)
3.	ATLAS.ti coding	To identify various levels and forms of anonymity discussed in the data	1) *The need for strong anonymity*, effort to be untraceable; 2) *The need for some degree of anonymity*, effort to be unidentified: pseudonymous, faceless and/or voiceless; 3) *No need for anonymity* (this was left out of the detailed analysis, as we focus on the personal need for anonymity).
4.	Detailed interaction analysis	To develop a nuanced understanding of A) the connection between contextual relations and the need for anonymity and B) how the service users accounted for their needs for anonymity	The service users gave both excusing and justifying accounts of their needs for anonymity, with the former being more common. We connected these accounts to the following contextual relations: 1) *Excusing the need for anonymity by referring to societal relations*: blaming Finnish society for stigmatising attitudes and exclusionary practices 2) *Excusing the need for anonymity by referring to service system relations*: blaming the service system for the risk of negative consequences due to recording the use of illicit drugs 3) *Justifying and excusing the need for anonymity by referring to personal relations*: appealing to personal situations, feelings and experiences The Results chapter provides detailed analyses of the service users’ accounts under the subheadings indicated above. The first part of the heading represents the general connection between wider relations and the service user’s way of giving accounts. The second part reflects how this connection is concretised in the service users’ talk. The relations mentioned in the subheadings were generally reflected in the data, but they intertwined situationally with other relations (societal, service system, personal, spatial and time relations) in each service user’s talk.

In analysing the explanations of the need for anonymity, we apply the concept of *account* [22]. The analysis of accounts has a well-established position and long history in ethnomethodological research that is focused on socially and culturally shared rules and conventions that people follow in their everyday interactions and on the ways of talking through which individuals construct a stable social reality [35]. Accounting refers to rationalising the contradiction between culturally expected actions (in this case, revealing personal data in services) and proven actions (in this case, protecting anonymity in services) [22]. People give accounts to make their actions appear consistent, understandable, legitimate and morally acceptable so as to avoid undesirable consequences [36].

Accounts can be produced to explain either an individual’s own or someone else’s actions [22, 37, 38]. Both types are present in the data of people explaining their need for anonymity. According to Scott and Lyman’s theory [22], which is widely used in empirical social science research [e.g. 37, 38], accounts can be either *excusing* or *justifying* ways of talking. All people give these kinds of accounts in their everyday lives, as accounting is an integral part of human interaction [38]. *Excusing accounts* shift the responsibility for needing anonymity (a culturally unexpected action) to other people, communities or factors related to the situation, which can manifest as *blaming*. In these accounts, people indicate that they had unconditional personal reasons for deviating from cultural expectations (i.e. the expectation of revealing their personal data). *Justifying accounts* involve taking personal responsibility for the need for anonymity. This culturally unexpected action is also seen as important and reasonable in certain situations or circumstances [22, 36–38].

When analysing accounts, we do not imply value judgements on anyone, particularly people who use drugs and who face considerable perceived and actual stigma [39]; instead, we use the conceptual framework of analysing accounts as a tool to illustrate the rationale. We aim for a systematic analysis of service users’ talk to identify both shared and individual logics in how they account for the need for anonymity. We see accounting as a result of wider relations (such as established logics or values in a given society or service system), which create situations in which accounts of the need for anonymity are culturally expected. Thus, we do not expect service users to account for reasons attributable to themselves.

## Results

All the service users identified needs for social and healthcare services. Most described applying for services as requiring a certain form of anonymity as accounted for under a wide variety of themes, the most common being mistreatment caused by societal stigma (Table 3).

**Table 3:** Themes emerging from the needs for anonymity of 28 service users

Theme	Number of mentions in the data
Mistreatment caused by societal stigma	22
Records in shared social and healthcare information systems	20
Loss of face; shame or guilt over one’s own situation or change in people’s attitudes	15
Losing a job or place of study	11
Inability to access physical services due to personal reasons	7
Consequences for family and/or children	5
Being left without necessary medication	4
Loss of trust in society or other people	3
Discontinuation of treatment in mental health services	3
Criminal liabilities	3
Consequences for driving license	1
**Total**	**94**

### Excusing the need for anonymity by referring to societal relations: blaming Finnish society for stigmatising attitudes and exclusionary practices

*Hi. First*,* thanks for this thread. One dares to be honest here*,* unlike with doctors in Finland*,* thanks to politics.* (…) *Now I have this good job that I like*,* but on the other hand if I ask help from somewhere I’ll probably lose the job because it’s drugs instead of booze*,* then I would even get some help* ((in the case of alcohol)). *It’s a shitty thing in this society that you must hide your need for help and try to fix yourself with the help of a street doctor. Sigh*,* one day at a time. Yeah*,* yeah*,* still a ((drug)) user but let’s at least try.* (DWBS, online discussion A)

This excusing account includes reflection between societal relations and service system relations. The service user blames Finnish society by describing how its “*politics*” (e.g. drug policy based on criminalisation, which defines people who use drugs as ‘criminals’) lead to hiding drug use from healthcare professionals. Thus, we interpret the first “*thanks*” as genuine and the second as sarcastic. The forum providing anonymity is defined as a place where people can talk honestly about drug use, thus connecting societal and spatial relations. Finnish society is also blamed for drug exceptionalism; that is, alcohol is treated differently from other drugs. In another excusing account, the responsibility for needing anonymity is again shifted to society due to fear of the exclusionary practice of terminating employment. “*It’s a shitty thing in this society*” strengthens the argument that it is Finnish society that prevents people from being honest and leaves people alone in need of help. According to an interview with another DWBS user, societal stigma prevents people from asking for help under their own identities:

*SERVICE USER: The attitude ((towards people using drugs)) should certainly change. It’s just that no one really enjoys where they are at the end of the day*,* at least I don’t enjoy it myself. Maybe even the escalation of my own personal situation ((refers to initiation of intravenous drug use)) could’ve been prevented by being able to talk about those things more openly and at a very early stage.* (…)

*RESEARCHER: Would you go*,* if we imagine that there was anonymous rehab and rehabilitation available in Finland*,* would you go*,* or would it feel easier?*

*SERVICE USER: I would*,* absolutely would ((go)). I would’ve gone a long time ago.* (DWBS, interview 2)

In this excusing account, the responsibility for the need for anonymity is shifted to other citizens by demanding a change in societal attitudes. The blaming tone is strengthened by humanising perceptions of people who use drugs and who may be suffering in their current life situations. The service user describes how avoiding stigma has forced them to hide their personal situation and delay seeking help, illustrating how societal relations intertwine with service system relations. Indeed, if anonymity in face-to-face treatment was available, this service user would have asked for help earlier and taken the risk of facing societal stigma.

The topic of criminality was discussed in some interviews. One SBS user (interview 12) gave an excusing account by blaming the current drug legislation and expressed hope that drug use would be decriminalised in Finland. This wish was directly connected to service system relations, i.e. to barriers in seeking help from needed services: “*You can’t seek help for the problems that you could otherwise seek because of the fear that you will also get caught in it yourself and be a criminal*”. Another SBS user described how, among other things, criminality can be an obstacle to equal membership in society:

*SERVICE USER: You have to remember people’s aversion to drugs*,* many of them feel disgust. Then the fact that you’re breaking the law*,* it’s also a legal case if you use drugs. It involves a lot of issues.*

*RESEARCHER: Many reasons.*

*SERVICE USER: You won’t be accepted into any job*,* you won’t be accepted into any school*,* you won’t be accepted into any rehabilitation work programme. It’s a lot to take in. In a way*,* you’re left completely stranded. That’s why anonymity is important in a service like this.* (SBS, interview 8)

By using the strong expressions “*aversion*” *and* “*disgust*”, the service user highlights society’s depth of stigma regarding drug use and people who use drugs, thus showing the difficulty of accessing services if one has a known history of drug use. The service user connects the excusing account to Finnish drug legislation, one of multiple elements that makes anonymity necessary under current societal relations. We interpret the described scenarios of exclusionary practices as excusing accounts of the need for anonymity. They blame society for negative consequences for individuals if their drug use comes up in services; people will be rejected from work or school and denied opportunities. Describing these possible scenarios as facts stresses that they are experienced as serious risks. The service user argues that, due to these exclusionary practices, there will be nothing left in the end: “*In a way*,* you’re left completely stranded.*” A similar experience was reflected by another SBS user (interview 13): “*They ((acquaintances who use drugs)) feel that society has completely left them. So*,* you don’t have the nerve to ask for help anymore*.”

### Excusing the need for anonymity by referring to service system relations: blaming the service system for the risk of negative consequences due to recording the use of illicit drugs

*SERVICE USER: I can’t imagine contacting health or social care because of the social consequences. (…) Once there is a record in healthcare about drug abuse*,* that stigma follows you forever.* (DWBS, interview 1)

“*I can’t imagine*” reflects the impossibility of contacting public services in their current circumstances. The risk of “*social consequences*” —later related specifically to being a single parent—explains the need for strong anonymity in the DWBS. The blaming tone in this excusing account is evoked when the service user describes the irreversible consequences should the drug use be recorded even “*once*” in the shared information system. This highlights the bond between the service system and time relations; according to the service user, one drug-related record in a history can define a future permanently, “*forever*”, even if people stop using drugs. The service user confirms the risk of stigmatisation by judging that a person who uses drugs and is a single parent will be treated in an unwanted way.

In contrast, contacting the DWBS “*facelessly from behind the pseudonym*” offered a surprisingly positive experience. The same service user (interview 1) later described how the DWBS worker “*took my situation seriously*,* (…) I received contact information and advice*”. The absence of judgement strengthened their trust in the DWBS: “*And nobody has judged at any point*”. The fear of being stigmatised was overcome, firstly, by offering technological anonymity. The positive experience of interaction was possible only online, connecting the need for anonymity to spatial relations, and secondly by the respectful encounter, referring to personal relations, i.e. their confidential relationship with the HR worker.

The fear of stigmatisation in healthcare is also present in the SBS interviews:

*SERVICE USER: I didn’t know you could call anonymously on the phone and order ((injection)) equipment at home. As I think it’s very important that you don’t get these ((infections))*,* I’ve used the same old shitty needles a lot*,* then I’ve got some infections. People can’t afford to buy medicine*,* and they don’t have the guts to go to the doctor*,* and the doctor looks at you disapprovingly*,* like when are you going to quit. Or that information will go to an opioid substitution treatment clinic or something*,* so people will not go and treat them ((infections)). It’s very important that they ((clean needles)) are shared*,* and the information about this is spread so people would dare to call ((to the SBS)) more often.* (SBS, interview 9)

This excusing account includes blaming the service system, as the service user describes the obstacles people often face when they aim to empower themselves to reduce drug-related harm. The options are either to continue using “*shitty needles*” or to take the risk of encountering doctors with a moralising tone, which would also compromise their treatment pathway, i.e. cause sanctions in opioid substitution treatment. The service user places the responsibility for needing anonymity on the service system. Thus, offering HR services that protect anonymity can be the only way to obtain clean needles and avoid infections instead of using dirty ones. The service user defines as important factors the pseudonymous and faceless contact by phone, which draws attention to the importance of spatial relations in services.

The fear of information spreading in the system was present in many interviews. One SBS user (Interview 8) stated in an excusing account that recording drug use in a shared system causes quite definite consequences: “*It’s pretty sure that information will also go forward if it goes to that point*”, and, eventually, “*it always results as sanctions from somewhere*”. The service user does not clearly specify what they mean by saying that personal data will “*go forward*”, but the statement reflects a serious concern about information spreading to the wrong parties, which would result in negative personal consequences. In contrast to this blaming, the service user gives credit to the SBS, which secures anonymity and thus makes honesty much easier: “*Here I can talk honestly about things as they actually are*.” The following DWBS user mentions a similar pressure to hide drug use:

*SERVICE USER: Yes*,* you have to hide it ((the drug use)) and specifically*,* you can never be completely honest. In a way*,* you know that you must lay everything on the line at the point where you go to ((an outpatient addiction treatment clinic)) or a psychiatric outpatient clinic to seek help for a long-term drug problem. That is exactly the thing*,* it can at worst overshadow the rest of your life. Then if one day there was a need for certain services or recipes*,* you won’t get them*,* due to the background of problematic drug use. I could go so deep into that*,* you know*,* I don’t personally know any drug addict who would’ve started using drugs for fun*, *you always have something heavy in the background.* (DWBS, interview 2)

The service user describes the combination of needing help and problematic drug use as leaving one no choice when trying to get help; you put your whole life and potential future at risk. This blaming, excusing account lays on the service system the responsibility for this either/or situation, perceived as unreasonable and counterproductive to making life improvements to sustain needed changes. The need for anonymity becomes evident when records of a long history of drug use are deemed to have permanent negative consequences, connecting service system relations to time relations: *“It can at worst overshadow the rest of your life*”. The service user gives concrete examples: in the future, needed medicine would not be prescribed, or services would not offer needed support. The responsibility is shifted to the service system instead of to individual people; in this account, the interviewee states that individuals whom they knew did not start using drugs “*for fun*” but to alleviate negative experiences.

Later, this service user (interview 2) describes not being categorised as “*officially a drug addict*” in healthcare records, which has allowed them to avoid the stigma that others experience. The service user reflects that stigma is the pivotal factor preventing them from accessing any health services and that it motivated their contact with the DWBS. Risking one’s own health due to fear of stigma in public services resulted in the initiation of intravenous drug use, which inspired an excusing account with a blaming tone. In this account, it is the service system’s fault that the situation with drugs worsened: “*I’ve been trying to protect it ((own identity)) to the last*,* even at the expense of my own health*.” Due to societal stigma, technological anonymity offers the only possibility of seeking help from services, which binds spatial, societal and service system relations to each other. The service user also describes how revealing drug use could affect long-term mental health treatment:

*SERVICE USER: Given the length of time I’ve been using hard drugs*,* it’s possible that psychiatric and other healthcare services will begin to interpret everything I’ve shared about my life and attribute it to drug use.* (DWBS, interview 2)

The excusing account indicates why this service user needs anonymity: there is a risk that if mental health services discover the drug use, the service user’s life story and identity, constructed during this treatment, would be changed from a person who needs mental health treatment to a person who is addicted to drugs. The responsibility for needing anonymity is shifted to the service system, which is blamed for reconstructing the service user’s life story based on drug use rather than examining it as presented to the service system. Thus, strong anonymity is necessary in the current service system to enable maintaining one’s own identity, getting needed help from the mental health service and seeking help for drug addiction from another service (DWBS).

### Justifying and excusing the need for anonymity by referring to personal relations: appealing to personal situation, feelings and experiences

*SERVICE USER: But of course*,* it ((the need for anonymity)) is probably quite situational. For me*,* it was like the loss of face and shame. But then*,* some people may have been committing crimes and so on*,* so they have*,* in a way*,* a lot more to lose. I think it might be more specific for them how much to tell and to whom.* (DWBS, interview 3)

The service user mentions individual, “*situational*”, differences in the need for anonymity; people who engage in criminal activity may have much “*to lose*”, so they have a special need to assess what information they share and with whom. In contrast, the service user’s personal need for anonymity is based on negative feelings, described as “*the loss of face and shame*”, which the service user later associates with family. According to this justifying account, the service user does not want to experience heavy feelings or to hurt the feelings of close ones, so anonymity is needed. The latter also reflects personal responsibility regarding the need for anonymity. When asked whether it mattered to them that they did not have to meet the DWBS worker face to face to receive help, the service user responded as follows:

*SERVICE USER*: *It does matter in the beginning. I feel it has been quite important in the beginning. But I don’t know*,* it’s somehow exciting how trust can be built this way*,* too. But in my situation*,* it’s like I’ve decided that I’m going to do everything I can to get things done. So*,* for example*,* while I’ve been talking here ((in the DWBS))*,* it feels like the need for anonymity has disappeared a bit during this time.* (DWBS, interview 3)

The service user again reflects the situational nature of anonymity; protecting identity is especially important when applying for services for the first time, before a confidential relationship is built. The personal need for anonymity diminishes as trust in the DWBS worker increases over time, indicating that personal relations are intertwined with time relations. Their justifying account includes taking personal responsibility for reducing the harm of drug use, and anonymity is needed in this specific situation. Later, the service user reflects that anonymity increased the motivation to take personal responsibility for changes to their drug use: “*I’ve noticed that an anonymous encounter on the internet has given strength to*,* like*,* it shows that someone understands you. (…) So now I’ve got some boost to think*,* like okay*,* I’m really going to try my best.*” A motivation to quit using drugs for personal reasons was also described in the DWBS online messages:

*I’m a second-year practical nurse student addicted to opioids. I’d like to ask for help but I’m afraid I’m going to get kicked out of the school as a result. Can a practical nurse be*,* for example*,* a client of a substitution treatment?* (DWBS, online discussion B)

The service user balances between the need for help with opioid addiction and the risk of losing their study place if their drug use were known. They deem that the “*school*” will take the view that it is not suitable to use drugs and be a practical nurse. “*I’d like to ask for help*” indicates personal motivation to seek treatment for drug use. Taking personal responsibility and showing personal motivation characterise a justifying account. Asking whether a healthcare professional can be a client of a substitution treatment requires strong anonymity, but a positive answer would solve the complex situation without a risk of personal loss; if the treatment were possible, the service user could even reveal their personal data. One SBS user also argued that it would not be a problem to record personal data if it were used only in a needle exchange unit or in the SBS. However, the service user was concerned that this data would be shared with other health services:

*SERVICE USER*: *And I think they ((in the SBS)) know my name*,* of course they know. And it doesn’t bother me. And the ((home)) address. It’s quite natural. But not like it is said in all systems that now this person visited ((the SBS)) to pick up needles. That it would be recorded in OmaKanta ((the national database for health data)). ’Cause all the information is there. And I don’t use any tranquillisers. I’ve been on blood pressure medication for another year. Then there’s the prostate medication. (…) But I don’t use any other medication. Well of course*,* there’s the HIV medication.* (SBS, interview 7)

This excusing account has a slightly blaming tone; health services should use only information that is relevant to them and have no right to access information about drug use if it is not necessary for their specific medication or treatment. The service user should have the right to self-determination and not to share information about drug use with other services beyond the SBS or needle exchange units. The service user has revealed their identity and home address in the SBS, and they do not see it as problematic. Instead, “*It’s quite natural*” as the SBS focuses on reducing drug-related risks, facilitating home visits for this purpose. The permission to access the home shows trust in SBS workers, reflecting the connection between personal and spatial relations. One DWBS user also connected personal health issues to the need for anonymity, but from a different angle:

*SERVICE USER: I’m a bit of a hypochondriac myself*,* so I’ve usually just made contact ((with the DWBS)) if I’ve been longing for a professional interpretation to a certain issue. If I haven’t had the opportunity to visit the local needle exchange unit or so on*,* then I’ve contacted them ((DWBS)).* (…) *And in fact*,* I suffer from very troublesome agoraphobia and panic disorder*,* the fear of social situations and anxiety*,* so I didn’t dare to visit there ((the needle exchange unit)) many times to get clean equipment*,* so they ((DWBS)) contacted the unit*,* and they delivered clean equipment to the area where I was.* (DWBS, interview 2)

In this extract, “*hypochondriac*” refers to the need to seek professional advice from the DWBS to rule out personal concerns related to health. The DWBS offers an option for anonymous contact when their mental health disorders— “*very troublesome agoraphobia and panic disorder*,* the fear of social situations and anxiety*” —prevent the service user from visiting needle exchange units. In this justifying account, the service user connects the need for anonymity to their personal health situation instead of to other people. This is again an example of the situational nature of the need for anonymity; the same person contacts both physical services and the DWBS depending on their current capacity for encountering people face to face. This underlines the connection between personal and spatial relations. One SBS user also discussed the difficulty of showing their own face:

*RESEARCHER: What does it mean to you that you can send a message and you don’t necessarily need to call?*

*SERVICE USER: It makes a lot of difference. I can’t talk on the phone at all. I mean I know it’s very hard for a lot of people to call. So*,* the fact that you can send a message*,* it’s a huge deal.* (…) *I don’t know what it is*,* something like shame*,* I guess. It’s so much easier to speak through a message. I don’t want to be a burden by asking for help or… Maybe it’s easier to dip a toe in a bit and send a message first.* (SBS, interview 13)

The service user describes that anonymity is not strong enough in person or even on the phone, which, according to their account, prevents them from accessing any services. The service user connects this justifying account about the need for anonymity to a personal experience of shame. However, describing being “*a burden*” to the services discloses the feeling of unworthiness that people who use drugs may experience due to self-stigmatisation. We interpret this as a result of other people’s negative attitudes. Protecting one’s face requires an anonymous first contact: a message through “*Wickr((Me))*” as the service user later elaborates. The service user describes this as especially important if personal feelings are very negative. The fact that *“you don’t need to reveal everything right away on the phone”* lowers the threshold for asking for help and enables determining whether the worker is a friendly and trustworthy person. The service user argues that this kind of anonymity is a key factor in building trust in this particular worker: “*Then I’d relax right away*”. This once again indicates the intertwining of spatial and personal relations.

## Discussion

In this article, we explored various meanings of anonymity in the context of dark web- and street-based HR services in Finland. We analysed what kinds of needs for anonymity the service users expressed when discussing seeking help to reduce drug-related harm, how they accounted for their need for anonymity when seeking such help and to which kinds of contextual relations these accounts were attached.

Our data made evident a broad and relational understanding of the concept of anonymity and the individual experience of being anonymous [see 27, 28]. According to our assumptions, the need for anonymity did not vary only between different individuals but also for individual participants depending on their current living circumstances and life situations. The service users made personal risk assessments of what they would lose if their drug use were recorded in the service system. They feared that the dissemination of information about an individual’s drug use could lead to unwanted repercussions, such as negative changes in treatment by healthcare providers, cessation of mental health services or cancellation of medication prescriptions. They also voiced worries about the effects on family members. Moreover, the risk assessment included the societal stakes attached to personal privacy, such as loss of educational or employment opportunities. In some cases, this was considered to be a permanent change that would affect life opportunities well into the future. This challenges the narrative in treatment that contends that individuals must adopt different identities to evolve from their current position [40]. It is challenging when people want to end their drug use in a society that will not let them fully move on from their drug use histories and escape perceived or experienced societal stigma. As others have noted, this also promotes social inequality and inequity in healthcare and may prevent some seeking help [41].

We connected the service users’ accounts of the need for anonymity to the following contextual relations: (1) excusing the need for anonymity by referring to societal relations, which was manifested in the service users’ talk as blaming Finnish society for stigmatising attitudes and exclusionary practices; (2) excusing the need for anonymity by referring to service system relations, manifested in blaming the service system for the risk of negative consequences due to recording the use of illicit drugs; (3) justifying and excusing the need for anonymity by referring to personal relations, which appeared in the data as appeals to the service users’ personal situations, feelings and experiences. The blaming accounts, in which the responsibility for needing anonymity was placed on Finnish society or the service system, indicate that anonymity was used as a defence against perceived abuses of power against these service users. This finding underlines that people who use drugs are not passive consumers of care; rather, accounting for the need for anonymity reflects active participation in defending one’s right to obtain needed help. Thus, these accounts were motivated by rational action to avoid possible sanctions in society and in services, which were assessed to have negative consequences for the service users’ lives.

This study aimed to give space to the experiences and thoughts of HR service users in Finland. Because this ethnomethodological study analysed service users’ ways of accounting for their situational needs for anonymity, this research does not assess whether the risks reflected in the service users’ talk are based on ‘right’ or ‘wrong’ facts. However, it is important to note that identical kinds of accounts and reasons for needing anonymity were repeated in multiple service users’ talk. This indicates either that these factors have the potential to become real risks in Finland or at least that the perception of these possible consequences is culturally shared among people who use drugs. Therefore, it is worth considering how these widely shared fears could be reduced culturally and in practice.

Our analysis reveals that the reasons for needing anonymity are often fundamentally based on shared societal relations: the cultural stigma towards people who use drugs [see 39]. In line with previous studies, mistreatment caused by stigma was seen as an insurmountable barrier when seeking help from services [see 3–5], which inspires much concern. The severity of this risk is reflected in another Finnish study that analyses the decisions of the Parliamentary Ombudsman; the researchers characterise this kind of mistreatment as ‘useless suffering’ that services may cause among people who use drugs [42]. According to our study, the only way for some to avoid this risk was to remain anonymous. In addition to pseudonymity, the service users often paid attention to meeting places. For example, if they could not encounter HR workers in person in public places, they contacted the workers through online platforms that provided strong anonymity [see 12]. Considering this, HR services that provide the opportunity to contact them both in person and online seem essential.

Our findings suggest that trust (or its absence) influences the need for anonymity [see 20]. Our observations raise critical questions about the role and necessity of anonymity, both online and in physical encounters. If (service) systems could guarantee privacy through trust-based mechanisms—such as not recording information or legally prohibiting data transfer—the need for anonymity might be obviated. This challenges the binary perception of anonymity [see 28], highlighting its nuanced nature and suggesting that in environments where confidentiality is assured, the negative outcomes typically associated with information disclosure may not occur, reducing the need for strong anonymity. The need for anonymity was also related to confidential personal relationships, i.e. trust-building practices in encounters between service users and HR workers. Anonymity was especially important at first contact, but the need for it diminished as trust in HR workers strengthened. Trust was built by non-judgemental and pragmatic interactions, based on the human rights principles of HR [11], that respected the individual seeking help [see 12].

Because of this article’s focus on examining ways of producing accounts in service users’ talk, it has not considered the individual characteristics of service users or their social status in relation to the dominant society. Future studies should therefore explore the relationship between these factors and the need for anonymity. In addition, the limited number of research participants may not accurately reflect the experiences of all people who use drugs. However, we found many kinds of need for anonymity in different contexts of HR. Thus, we argue that our data were versatile and provided a broad, nuanced picture of various meanings of anonymity. In addition, our aim was to obtain a situational picture of the Finnish case. Therefore, despite communalities in drug policy, societal views and stigmas, we cannot generalise the results to all societies. Nevertheless, the results show that there are people who benefit from anonymity when seeking help to reduce the harm of drug use, and it is worth considering whether the individuals’ anonymity should be more widely and strongly protected in countries that prioritise criminalisation in drug policies.

This study confirms that the concept of relational anonymity provides an important understanding as well as tools for promoting the social rights of individuals who use drugs in societal relations in which drug use is strongly stigmatised. We suggest analysing this topic in other countries to determine whether the need for anonymity is connected to the same kinds of concerns as in Finland. In addition, the HR community would benefit from knowledge regarding the need for anonymity in countries where drug use is decriminalised or legalised.

## Conclusion

HR services use the practice of anonymity it in a variety of ways during face-to-face and online meetings as well as on the open web, the Tor network and instant messaging applications that enable anonymity. Our study shows the importance of recognising the ambiguity of the concept in future HR services, as the strength of the offered anonymity is not irrelevant. In our data, the service users’ active consideration of the possible risks associated with recording drug use in the shared information system reflected the significant importance of anonymity. Sometimes, this reflection was quite broad, and the indication of whom the information would eventually spread to was imprecise, which may indicate a lack of factual information on this topic. Thus, service users should be informed about the meaning of anonymity in certain services, the extent to which their privacy is protected, which personal data are recorded (or not), who can read the data and how to restrict the use of their data. Services should be able to provide information on what consequences specific records can and cannot cause, as people worry that records about drug use will be permanent and that their current or past choices (such as accessing needed services) will impact their future lives.

In our interview data, needing anonymity was clearly more common than not needing it. This indicates that although sharing health information in different services has legitimate aims, its negative consequences should be seriously considered; it is not appropriate that people avoid using public services for as long as possible. According to our data, HR services offering anonymity were often the only services from which service users received help in reducing the harm of drug use. This is also an important counterargument to the categorisation that people using drugs are ‘hard to reach’ in services [e.g. 43–44]. From the point of view of those who use drugs, it is the system requiring the disclosure of personal information that can make the service difficult or even impossible to reach. Service user involvement in service development should mirror other areas of healthcare [45, 46], even with the understanding that service providers and service users may sometimes not fully agree [47]. Our results underline that the need for different forms of anonymity extends beyond future HR services to encompass entire service systems. Securing anonymity even in the first encounter, enabling the process of building trust to begin, is important in health and social services in general.

As our study makes clear, the need for anonymity does not arise separately from its current circumstances [see 23]. The need for anonymity indicates problematic societal relations in which the stigma on drug use is still very strong. In the future, societies should cultivate cultural atmospheres and information sharing practices that eliminate the need for anonymity. However, under current societal conditions, HR services that protect anonymity in various forms are crucial. It is alarming that people feel that they are left alone with their (potentially problematic) drug use, which may bring even life-threatening risks. The indivisibility of human dignity and the equal treatment of people are fundamentally established in the UN’s Universal Declaration of Human Rights [48]. Therefore, society should bear more responsibility for changing attitudes, rather than individuals in need of assistance.

## Data Availability

The interview data, collected privately, are not publicly available; we aim to protect individuals’ anonymity. The naturally occurring data (online conversations in the Torilauta discussion forum) are available for restricted use via the Language Bank of Finland (http://urn.fi/urn: nbn: fi: lb-2022062221).

## Footnotes

1 The messages analysed in the article were selected from all messages sent to the Torilauta online forum during the periods 11.9.2019–20.5.2020 (1,859,121 messages in 251 days) and 17.6.2020–31.10.2020 (1,099,708 messages in 136 days) as well as 146,147 messages sent outside these periods, which remained on the site during the collection (including DWBS discussions from 2018 onwards). The analysed sample consisted of message threads that mentioned one or more DWBSs by name or employee nickname. The data were collected and provided for research purposes by the Torilauta site administration.

## Abbreviations

DWBS: Dark web-based service
HR: Harm reduction
NA: Narcotics Anonymous
SBS: Street-based service
